# Built Environment Influences of Children’s Physical Activity: Examining Differences by Neighbourhood Size and Sex

**DOI:** 10.3390/ijerph13010130

**Published:** 2016-01-15

**Authors:** Christine A. Mitchell, Andrew F. Clark, Jason A. Gilliland

**Affiliations:** Department of Geography, Western University, 1151 Richmond St., London, ON N6A 5C2, Canada; cmitch57@uwo.ca (C.A.M.); aclark2@uwo.ca (A.F.C.)

**Keywords:** built environment, accelerometer, GIS, physical activity, neighbourhood, child

## Abstract

Neighbourhoods can facilitate or constrain moderate-to-vigorous physical activity (MVPA) among children by providing or restricting opportunities for MVPA. However, there is no consensus on how to define a child’s neighbourhood. This study examines the influence of the neighbourhood built environment on objectively measured MVPA among 435 children (aged 9–14 years) in London (ON, Canada). As there is no consensus on how to delineate a child’s neighbourhood, a geographic information system was used to generate measures of the neighbourhood built environment at two buffer sizes (500 m and 800 m) around each child’s home. Linear regression models with robust standard errors (cluster) were used to analyze the relationship between built environment characteristics and average daily MVPA during non-school hours on weekdays. Sex-stratified models assessed sex-specific relationships. When accounting for individual and neighbourhood socio-demographic variables, park space and multi-use path space were found to influence children’s MVPA. Sex-stratified models found significant associations between MVPA and park space, with the 800 m buffer best explaining boys’ MVPA and the 500 m buffer best explaining girls’ MVPA. Findings emphasize that, when designing built environments, programs, and policies to facilitate physical activity, it is important to consider that the size of the neighbourhood influencing a child’s physical activity may differ according to sex.

## 1. Introduction

Obesity rates among Canadian children and adolescents have risen dramatically over the last thirty years, in part due to decreasing levels of physical activity [1,2,3]. Obesity is a complex health problem with numerous mechanisms, but it is generally agreed that obesity is the result of an energy imbalance that occurs when the energy consumed exceeds the energy expended [4]. Physical activity increases energy expenditure and therefore helps prevent obesity [5]. Regular physical activity during childhood also helps to mitigate risk factors associated with cardiovascular disease and improve psychological well-being by improving academic performance and reducing anxiety and depression [6,7]. Yet, few Canadian children are meeting Canada’s recommended physical activity guidelines of at least sixty minutes of moderate-to-vigorous physical activity (MVPA) during most days of the week [8,9]. Canadian children now spend the majority of their time engaging in sedentary activities like watching television or playing on the computer [8]. 

Physical activity is a complex behavior and there is growing interest in ecological models of health to explain how a diverse range of mechanisms influence physical activity at multiple levels ranging from intrapersonal (e.g., age, sex, preferences, attitudes), interpersonal (e.g., household income, parental education, parental occupation), environmental (*i.e.*, built and natural), and policy [10]. At the intrapersonal level, boys tend to be more physically active than girls, with recent research finding that 13% of boys aged 5–17 and only 6% of girls aged 5–17 meet Canada’s recommended physical activity guidelines [8,11]. Research has found that girls prefer different activities, have different motivations for being physically active, and face different barriers to physical activity than boys [12,13]. For example, boys have more independent mobility providing them greater access to opportunities in their neighbourhood [14,15]. In addition, the mode a child uses to travel between home and school has been found to contribute to a significant proportion of their overall physical activity levels. Children who use active modes of travel between home and school (*i.e.*, walking or biking) tend to be more physically active and are more likely to meet daily MVPA recommendations than those using inactive modes [16].

At the interpersonal level, physical activity levels have been found to be lower among certain ethnic/racial groups (e.g., black children and youth) and lower socio-economic classes [17,18,19]. It is hypothesized that these groups experience unequal access to physical activity opportunities in their neighbourhood, which in turn may affect whether or not they engage in physical activity [17,20]. At the environmental level, the built environment can facilitate or constrain physical activity by providing or restricting opportunities for physical activity [21,22,23,24]. The neighbourhood opportunities for physical activity may be particularly important for children and youth due to extrinsic constraints on their independent mobility (e.g., parental rules, too young for a driver’s license), which typically limit their activities to locations that they can access by walking or biking [25].

Land use patterns, transportation infrastructure, and urban design have been conceptualized as built environment correlates of physical activity [23,26]. Land use patterns affect the distribution of opportunities for physical activity, such as the presence of neighbourhood park spaces [26]. Land use mix is frequently used because it is able to characterize complex land use patterns in one measure [23,27]. Transportation infrastructure affects how well children are connected with facilities and also affords a site for physical activity, such as intersection density [23,26]. Intersection density (*i.e.*, connectivity affects the route options available in a neighbourhood and better connected neighbourhoods have been hypothesized as being easier to traverse [28,29,30]. Urban design affects the appearance and arrangement of physical features within spaces, such as recreation facility and park design or quality [23,26,31,32]. 

There is a need to explore the role of neighbourhood size because there is little agreement regarding how to best define a child’s neighbourhood [27]. Both buffer-based measures and administrative units have been used to define a child’s neighbourhood. Although many accelerometer-based studies of the built environment-physical activity relationship use a buffer size of 800 m or 1000 m [31,33,34,35], some have used home-based buffers as small as 200 m [36] and as large as 2 kilometres (km) [32,37]. Different buffer sizes capture different environments and the most relevant buffer size differs according to the environmental context, the behaviour of interest, and the group being studied [27]. It is important, then, to consider and conceptualize the neighbourhood built environment at different sizes and examine what best defines a child’s neighbourhood. 

Few studies have examined the role of neighbourhood size, particularly with objectively measured physical activity and objectively measured environment contexts. Van Loon *et al*. [36] examined associations between the neighbourhood built and social environment and MVPA using 200 m, 400 m, 800 m, and 1600 m buffer sizes and found that the largest buffer size best explained MVPA compared to smaller buffer sizes. Prins *et al.* [32] investigated relationships between availability of parks and sports facilities and MVPA using 400 m, 800 m, and 2000 m buffer sizes and found no associations between objectively measured availability of facilities within different buffer sizes and objectively assessed MVPA. Both studies failed to distinguish between weekdays and weekend day physical activity. Children’s physical activity may differ during weekdays than on weekends, and similarly, children’s physical activity may differ during weekday school hours compared to out of school hours. Examining non-school hour physical activity is important to separate the impact of the neighbourhood built environment from school activities. The contexts used when calculating MVPA may affect physical activity outcomes and, thus, the relationships between physical activity and the built environment. 

As a result, this research has three main objectives: (1) to examine whether the opportunities present in children’s neighbourhood built environments influence objectively measured average daily MVPA during weekdays outside of school hours; (2) to assess if there are sex differences when examining whether neighbourhood built environment opportunities influence MVPA; and (3) to assess whether the conceptualization of neighbourhood size affects associations between the built environment and MVPA.

## 2. Methods 

This study draws data from a multi-year study called the Spatial Temporal Environment and Activity Monitoring (STEAM) project to investigate the effects of the built environment on health related behaviours of children aged 9–14. The STEAM project had two data collection periods (8 days in the spring and 8 days in the following fall) for each year, 2011–2013 inclusive. Only data from the spring collection phase was used in this study. All children were required to have parental informed consent for inclusion before they participated in the study. In addition, all children with parental informed consent were required to provide their personal assent to participate in the study. This study was conducted in accordance with the Declaration of Helsinki and the Canadian Tri-Council Policy Statement: Ethical Conduct for Research Involving Humans, and the protocol was approved by the Non-Medical Research Ethics Board of the University of Western Ontario (NM-REB #:17918S) and the respective research officers and/or committees of the participating school boards. 

During the study period, participating students from 34 elementary schools across the four school boards within the region completed an 8-day multi-tool procedure to record their neighbourhood activities, mobility, and environmental perceptions. Participants completed detailed daily activity diaries, wore portable accelerometers during all waking hours for up to 8 days, wore portable Global Positioning System (GPS) monitors during all waking hours for up to 8 days (GPS data were used only to determine the home location of each child), and both children and parents completed detailed surveys about demographics and their child’s neighbourhood behaviours and perceptions.

The sample used in this study is a subset of a larger sample (*n* = 851) of children from 34 schools in London and surrounding area who had demographic data from the child and/or parent surveys, had valid physical activity data in the spring, and lived and attended school in London, Ontario. Of the 851 participants in the larger STEAM sample, 101 were excluded from this analysis because they were part of the pilot year (2010), which used different accelerometer calibration methods than non-pilot years. A further 226 participants were excluded because they did not live within the city limits of London, Ontario. Participants were excluded from further analyses if they had fewer than two valid weekdays of accelerometer data (*n* = 41). Participants were excluded if demographic data from the child or parent surveys was unavailable (*n* = 48), resulting in a final sample size of 435 students with both objective neighbourhood built environment data and physical activity data. The 435 students come from 20 schools spread across the city of London in urban and suburban settings of varying socio-economic status.

### 2.1. Measures

#### 2.1.1. Physical Activity

Physical activity was measured using Actical Z accelerometers (Philips Respironics, Murrysville, PA, USA) with 30-s epochs worn on the hip. Participants were asked to wear the accelerometer for 8 consecutive days (including 4–6 weekdays) during all waking hours, only removing it for sleeping, bathing, and swimming. Participants were required to have at least 2 valid weekdays of data to be included in analyses, a common practice for analyzing children’s accelerometer data [38,39,40,41]. A valid day was defined as at least ten hours of wear. Motionless bouts (extended periods of zero counts) of 60 consecutive minutes or longer were considered non-wear time and excluded from analysis. A valid day has been defined as 8 to 10 h of wear time in previous studies of children’s physical activity [42]. Cut-points for children classified the accelerometer data and defined the threshold at which the data would be categorized as moderate-to-vigorous (>1500 counts/min) [43]. Differences in individual school and recess start and end times were accounted for in the analyses. The number of minutes spent in MVPA during non-school hours for each valid weekday was averaged over the total number of valid weekdays observed to calculate average daily MVPA for weekdays during non-school hours. To determine the average time spent in MVPA during other time blocks (*i.e.*, during class time, recess, all weekday hours), the number of minutes spent in MVPA during those specific time blocks were averaged over the total amount of valid days observed.

#### 2.1.2. Independent Variables

Following the ecological model of health, this study uses three levels of independent variables: intrapersonal; neighbourhood socio-economic status (SES); and the neighbourhood built environment (Note: policy is not considered as a variable in this analysis because it is the same across all participants). 

Individual level variables were used to account for factors specific to each child that may influence their physical activity. These variables used include (with the reference category italicized): sex (male versus female); age in years (continuous); the most frequently used mode of travel to and from school during a normal school week (active (mostly walk or bike) versus inactive (mostly car or bus)); and the presence of a sibling (only child versus has sibling versus prefer not to answer). The variables used were collected from multiple sources, including child surveys, parent surveys, and data recorded for each child when calibrating their accelerometer. 

Median family income (CAD) was used as a measure of the neighbourhood SES and used as a control. Median family income was defined as the area-level SES in the census dissemination area in which their home is located. Neighbourhood SES can act as a proxy for other household demographic variables such as parental education and occupational status.

The neighbourhood built environment was objectively measured using a Geographic Information System (GIS) (ArcGIS 10.2, Environmental Systems Research Institute, Redlands, CA, USA). Each child’s home addresses was identified by using the spatial means of their GPS tracks and then used as the centroid for all measures. The neighbourhood built environment was measured using two types of spatial analyses: (1) the shortest distance along the street network between home and specific activity sites for children (e.g., recreation centres and schools); and (2) multiple ring buffers (500 m and 800 m) around children’s home addresses. These buffer sizes were chosen because children are typically limited to the immediate area within which they are able (or permitted) to walk or cycle. Previous research has found that children at 12 years of age can walk up to 5 km/h [44], so an 800 m buffer is equivalent to about a 10 min walking distance and a 500 m buffer is equivalent to about a 6 min walking distance for an average child. In addition, previous evidence has found that boys have more independent neighbourhood mobility than girls [14,15], so using a 500 m buffer in addition to the 800 m buffer accommodates flexibility. Both 500 m and 800 m buffers have been used in previous studies exploring children’s neighbourhoods [24,31,35,45]. Euclidean buffers were used instead of road network buffers because some neighbourhood opportunity structures may not be captured due to a lack of road network access (e.g., a park or school yard).

After creating buffers around each child’s home, built environment measures were developed in order to characterize the neighbourhood opportunity structures within these areas. Existing research informed the selection of measures used to address a range of the hypothesized mechanisms influencing physical activity [23,26]. A description of the built environment variables used in this study and their definitions are found in Table 1. All of the environmental data were supplied by the Planning Division of the City of London (2014) [46].

**Table 1:** Description of the built environment variables included in this study.

Built Environment Variable	Description
Open space parks (#/km^2^)	The number of parks per square km within each buffer without any built recreational amenities.
Parks with at least one sports field (#/km^2^)	The number of parks per square km within each buffer containing at least one sports field (defined as tennis courts, soccer fields, baseball diamonds, and football fields).
Parks with at least one playground (#/km^2^)	The number of parks per square km within each buffer containing at least one playground.
Parks with both at least one sports field and playground (#/km^2^)	The number of parks per square km within each buffer containing at least one sports field and at least one playground.
Distance to the nearest school (km)	The shortest distance along the street network between each child’s home and the nearest public, Catholic, or private school in the City of London.
Distance to the nearest recreational site (km)	The shortest distance along the street network between each child’s home and the nearest arena or public/private recreational facility.
Land use mix	An entropy measure between 0 and 1 reflecting the distribution of land-use.
Multi-use path space (km^2^)	The amount of multi-use path area within each buffer.
Intersection count (#/km^2^)	The number of 3- and 4-way intersections within each buffer.

### 2.2. Statistical Analyses

Statistical analyses were performed with STATA SE 13 [47]. Linear regression models with robust standard errors (cluster) were used to analyze the relationship between average daily non-school MVPA during weekdays and attributes of the built environment. Selecting the cluster option accounts for observations that are clustered into groups (*i.e.*, elementary schools) and that these observations may be correlated within schools. Individual level and neighbourhood SES variables were included if bivariate analyses revealed a significant association with average daily MVPA during outside of school hours (*p* < 0.10). Several of the variables were skewed and transformed using either logarithmic or square root transformations. 

## 3. Results

### 3.1. Descriptive Statistics

Descriptive statistics about the sample can be found in Table 2 and Table 3. The majority of participants were between 11 and 12 years old (73.10%). Of the participants, 59.31% were girls and 40.69% were boys. Most participants had a sibling (80.92%) and used an inactive mode of travel between home and school (63.22%). The median family income (in CAD) was $71,758.

Participants spent on average 63.98 min of MVPA per day during weekdays (Table 3). Boys engaged in 20.235 min more MVPA per day than girls during weekdays (in school and out of school). During class time, boys engaged in 5.168 min more MVPA than girls, a significant difference. During recess time, boys engaged in 9.701 min more MVPA than girls, a significant difference. On average, participants spent 30.361 min per day in MVPA outside of school hours; boys spent significantly more time on average in MVPA outside of school hours than girls.

**Table 2:** Descriptive statistics for average daily minutes of MVPA (*n* = 435).

Variable	*n*	%
Age (years)		
9	10	2.30
10	69	15.86
11	187	42.99
12	131	30.11
13	36	8.28
14	2	0.46
Sex		
Boy	177	40.69
Girl	258	59.31
Presence of a sibling		
Only child	54	12.41
Has sibling(s)	352	80.92
Prefer not to answer	29	6.67
Mode of travel		
Active	160	36.78
Inactive	275	63.22
	**Mean**	**SD**
Median family income in CAD (in thousands)	71.76	26.89

**Table 3:** Descriptive statistics for average daily minutes of MVPA by gender (*n* = 435).

Variable	Average Daily Minutes of MVPA During Weekdays							
	During Class Time		Recess		Non-School Time		All Weekdays	
	Mean	*p*-Value	Mean	*p*-Value	Mean	*p*-Value	Mean	*p*-Value
Sex								
Boys	19.432	0.000	23.680	0.000	33.783	0.009	75.985	0.000
Girls	14.264		13.979		28.014		55.750	
Total Sample	16.367	-	17.927	-	30.361	-	63.984	-

Note: Mann-Whitney U test.

### 3.2. Model Specification

A series of models were specified to assess associations between neighbourhood opportunity structures and children’s MVPA while accounting for age, sex, mode of travel, the presence of siblings, and neighbourhood SES (Table 4 and Table 5). Models were stratified according to sex because of anticipated sex differences in relationships, but a model using the entire sample was developed to detect smaller statistical effects with a larger sample size. 

### 3.3. Model Results

Model results assessing associations between built environment characteristics and MVPA are found in Table 4. At the individual level, girls and those using inactive modes of travel between home and school had significantly lower average daily MVPA during non-school hours. In contrast, students in the sample with a sibling had significantly higher average daily MVPA during non-school hours. Significant associations were found between average daily MVPA and the density of parks with sports fields and multi-use path area at both 500 m and 800 m buffer sizes. Despite using different buffer sizes, the 500 m and 800 m buffers in the full model yielded similar results, with the same significant variables and model fit. Variables were assessed for multicollinearity using Variance Inflation Factors (VIFs) because one assumption of ordinary least squares regression is the absence of high multicollinearity. No variables were found to be highly collinear, with a maximum VIF of 1.72. 

Sex stratified models were created to examine associations that may be unique to males and females (Table 5). Sex-specific associations were found at the individual level. Both boys and girls that used inactive modes of travel between home and school had significantly lower average daily MVPA during non-school hours than those using active modes; however, this was only significant for boys in the 800 m model. Boys with siblings had significantly higher average daily MVPA, regardless of buffer size. Median family income was positively associated with girls’ average daily MVPA in both 500 m and 800 m models. The model for boys’ average daily MVPA indicated significant a positive association with the density of parks with sports fields, and a significant negative association with the density of parks with playgrounds. Both 500 m and 800 m models had the same significant predictors, but the 800 m model exhibited a better model fit than the 500 m model. After accounting for several individual level variables and neighbourhood SES, the model for girls’ MVPA indicated significant associations between MVPA and the density of parks with sports fields. The density of parks with sports fields was only found to be significant in the 800 m model, not the 500 m model. The 500 m model had a slightly better fit than the 800 m model. No variables were found to be collinear, with a maximum VIF of 1.74. The sex-stratified models better explained the relationship between average daily MVPA outside of school hours during weekdays and the built environment.

**Table 4:** Results of full model assessing associations between environment characteristics by buffer size and average daily minutes of MVPA outside of school hours during weekdays (*n* = 435).

Variables	Regression Coefficients by Buffer Size			
	500 m ^a^		800 m ^b^	
	B.	*p*-Value	B.	*p*-Value
Age (years)	0.097	0.918	0.160	0.853
Sex (base: boys)				
Girls	−4.779	0.015	−4.973	0.007
Siblings (base: only child)				
Has sibling(s)	5.933	0.027	6.496	0.027
Prefer not to answer	2.858	0.507	3.207	0.430
Mode of travel (base: active)				
Inactive	−11.202	0.000	−11.255	0.000
Median family income in CAD (10^-3^)	0.033	0.496	0.021	0.696
Open space park: #/km^2^	−0.824	0.135	−0.060	0.956
Park with sports field: #/km^2^	0.929	0.016	2.653	0.020
Park with playground: #/km^2^	−1.721	0.070	−3.088	0.184
Park with more than one unique feature: #/km^2^	−2.645	0.063	−3.966	0.090
Distance to nearest recreational facility **^c^**: km	−2.094	0.102	−1.607	0.175
Distance to nearest school **^c^**: km	1.116	0.661	0.146	0.949
Land use mix (×10^1^)	0.002	0.998	−0.558	0.539
Multi-use path: m^2^ (10^-3^)	1.407	0.018	0.584	0.031
Road connectivity: # of intersections/km^2^	0.042	0.280	0.035	0.636
Constant	32.898	0.038	34.318	0.024

Notes: Table entries expressed as B values (unstandardized regression coefficients); **^a^** R-squared = 0.1695; **^b^** R-squared = 0.1675; **^c^** Street-network based measures.

**Table 5:** Results of sex-stratified models assessing environment characteristics by buffer size and average daily minutes of MVPA outside of school hours during weekdays.

Variables	Regression Coefficients by Buffer Size							
	Boys (*n* = 177)				Girls (*n* = 258)			
	500 m ^a^		800 m ^b^		500 m ^c^		800 m ^d^	
	B.	*p*-Value	B.	*p*-Value	B.	*p*-Value	B.	*p*-Value
Age (years)	−0.134	0.957	−0.119	0.957	0.339	0.700	0.261	0.753
Presence of a sibling (base: only child)								
Has sibling(s)	10.077	0.003	11.984	0.004	2.164	0.443	1.694	0.561
Prefer not to answer	4.575	0.589	8.412	0.282	−0.679	0.900	−1.409	0.780
Mode of Travel (base: active)								
Inactive	−8.275	0.089	−9.940	0.041	−11.65	0.000	−11.184	0.000
Median family income in CAD (10^-3^)	−0.054	0.554	−0.091	0.262	0.099	0.034	0.103	0.032
Open space park: #/km^2^	−1.370	0.256	−0.445	0.848	−0.424	0.459	−0.098	0.915
Park with sports field: #/km^2^	1.363	0.020	3.657	0.048	0.880	0.055	2.760	0.032
Park with playground: #/km^2^	−3.403	0.042	−8.082	0.026	−0.171	0.866	1.237	0.603
Park with more than one unique feature: #/km^2^	−3.941	0.106	−6.996	0.098	−1.651	0.271	−1.187	0.544
Distance to nearest recreational facility **^e^** : km	−3.754	0.190	−2.721	0.334	−1.499	0.358	−1.318	0.370
Distance to nearest school **^e^**: km	4.939	0.340	2.984	0.461	−0.789	0.780	−1.230	0.633
Land use mix (×10^1^)	−0.326	0.681	−1.564	0.158	0.414	0.567	0.407	0.682
Multi-use path: m^2^ (10^-3^)	1.421	0.822	0.770	0.072	1.257	0.091	0.358	0.326
Road connectivity: # of intersections/km^2^	−0.022	0.052	0.016	0.926	0.087	0.100	0.055	0.383
Constant	42.992	0.148	48.789	0.085	18.858	0.159	18.25	0.143

Notes: Table entries expressed as B values (unstandardized regression coefficients); **^a^** R-squared = 0.1616; **^b^** R-squared = 0.1796; **^c^** R-squared = 0.1961; **^d^** R-squared = 0.1895; **^e^** Street-network based measures.

## 4. Discussion 

This study examined whether the opportunities present in a child’s neighbourhood built environment predicted objectively measured average daily MVPA during weekdays outside of school hours by (1) the sex of the child and (2) neighbourhood size. Results show sex differences and neighbourhood size differences in associations between the neighbourhood built environment and children’s MVPA. 

### 4.1. Children’s Weekday Physical Activity: Overall and During School Hours

Boys engaged in significantly more daily MVPA than girls during weekdays, with boys achieving, on average, 20.235 more minutes of daily MVPA than girls. This finding is consistent with evidence finding that girls consistently achieve less daily MVPA than boys [8,11]. Although boys engaged in significantly more daily MVPA than girls, the girls in the sample averaged 55.750 min of MVPA across all valid days during weekdays, which falls just short of Canada’s recommended physical activity guidelines (>60 min per day). 

A similar pattern emerges when investigating children’s physical activity during school hours. Although both boys and girls participate in within-school physical activity (*i.e.*, Daily Physical Activity, physical education classes) where similar levels of MVPA should be achieved, boys, on average, engaged in significantly more MVPA than girls both during class time and recess time. This may be a result of girls participating in more passive activities like socializing, an activity popular among girls this age, instead of physical activity during both in-school physical activity and recess [13]. Recess, in particular, appears to be a significant contributing factor to MVPA during school hours. As a result, efforts should be made to develop programs that specifically target and engage girls in MVPA during recess times to increase the intensity of activity within this context. 

### 4.2. Children’s Weekday Physical Activity During Non-school Hours: Individual level and Neighbourhood SES Influences

In-school time MVPA only accounted for a portion of children’s physical activity, reinforcing the need to examine children’s MVPA outside of school time. Boys engaged in significantly more daily MVPA outside of school hours than girls, supporting the need for sex-specific models. This study investigated associations between built environment characteristics and children’s physical activity in two dimensions: child sex and neighbourhood size using two buffers. Findings from this study show sex differences between neighbourhood built environment opportunities and MVPA. This finding is consistent with Van Loon *et al*. [36].

One of the strongest predictors of MVPA was mode of travel between home and school for both sexes, although the relationship was stronger for girls. While both boys and girls who use inactive modes of travel to school engage in less MVPA than those using active modes of travel, girls who use inactive modes of travel engage in even less MVPA than boys who use inactive modes of travel. These findings suggest that girls achieve a majority of MVPA outside of school hours through mode of travel alone. Active transportation can contribute to a large amount of a child’s daily physical activity, so these findings emphasize the importance of encouraging children to use active modes of travel, particularly for girls [14,16]. 

Results from this study found that girls from higher income neighbourhoods are more likely to engage in MVPA. Although girls engaged in less MVPA than boys, those girls from more affluent neighbourhoods were more likely to be physically active than girls from less affluent neighbourhoods. These results suggest that policymakers and programmers should develop physical activity interventions appropriate for girls, especially girls from low income households. 

### 4.3. Physical Activity during Non-School Hours: Neighbourhood Built Environment Influences

Children from neighbourhoods with greater access to parks with sports fields and higher multi-use path area had significantly higher average daily MVPA during non-school hours. Neighbourhoods with greater access to sports fields afford opportunities for both structured (*i.e.*, sports teams) and unstructured (*i.e.*, playing with friends) physical activity. This diversity may engage more children in physical activity than a space solely designed for structured or unstructured physical activity [12]. Multi-use paths primarily afford the opportunity for unstructured physical activity, especially active transportation [48]. Significant associations did not differ by neighbourhood size in the model for all children. However, given that sex has been found to significantly influence MVPA both in this study and the literature, sex-stratified models are necessary to examine whether neighbourhood size and related findings are sex specific. 

Sex-stratified models revealed sex differences in significant associations and the most relevant neighbourhood size. The neighbourhood size that best predicted girls’ MVPA was 500m, smaller than the 800m neighbourhood that best predicted boys’ MVPA. This finding highlights that boys may have a wider neighbourhood to engage in MVPA with than girls. Coupled with the fact that more significant neighbourhood built environment relationships were found for boys, this study’s findings suggest that boys may have access to and engage in more neighbourhood physical activity than girls. This might explain why boys engaged in significantly more physical activity outside of school hours compared to girls; boys might be allowed by their parents to play more independently in their neighbourhood. Research has found that boys have more independent mobility than girls, granting them greater access to physical activity opportunities present within their neighbourhood [14,15].

Significant associations were found between average daily MVPA during non-school hours on weekdays and the density of parks within each buffer in the sex-stratified models, but these associations differed according to the recreational amenities present within the park. Both boys and girls from neighbourhoods with greater access to parks with sports fields were found to have significantly higher MVPA, emphasizing the importance of planning and developing recreational spaces designed to support physical activity for all children. In contrast, boys from neighbourhoods where park designs tended to be centered around playgrounds had significantly lower MVPA. Together, these findings suggest that boys engage differently with parks having sports fields than with parks having playgrounds. This may be a result of age; the boys are nearing early adolescence and may perceive playgrounds as spaces for socializing rather than physical activity. This may also be a result of unsupportive equipment; the playground equipment found at parks may not be challenging or complex enough for active play [49]. 

### 4.4. Limitations

Although some significant results were found, many built environment attributes showed no association with average daily weekday MVPA during non-school hours. Of the studies using objectively measured physical activity and buffer-based neighbourhood measures, several have found significant associations [31,36,50], but others have found no significant associations [51,52]. This study did not differentiate between specific physical activity contexts (e.g., sport activities, free play, active transportation); the primary objective was to examine overall physical activity. A more in-depth examination of different activity contexts may reveal more specific associations with the neighbourhood built environment. In addition, the lack of significant findings may be because neighbourhood proxies are unable to capture children’s direct exposure to their environments. Buffers are useful for helping to characterize a subject’s general neighbourhood opportunities, but are insufficient for assessing children’s actual exposure to different features in their environments and identifying the importance of difference contexts for physical activities. 

The use of GPS technologies in combination with acceleometry shows promise for assessing children’s real exposure to their environments [53]. Neighbourhood proxies, like buffers, rest on the assumption that all physical activity occurred within that area, which may explain why studies have yielded mixed results. The combination of GPS tracking alongside accelerometry, however, allows researchers to understand physical activity within the neighbourhood context but also outside of that context. This is particularly important because children are mobile and unlikely to spend all of their time within their neighbourhood, especially considering that more parents are now driving their children to structured activities [54,55]. While GPS technologies still face technological and financial limitations, the combination of GPS and accelerometry allows researchers to answer questions about where MVPA and sedentary activities occurred. Buffers are useful to answer questions about how neighbourhood built environments influence physical activity behaviours (including characteristics of places people choose not to frequent), but the combination of GPS tracking alongside accelerometry shows promise for assessing children’s real exposure to their environments. 

As this study draws from data collected during the spring, the physical activity that was measured will be specific to this season. A systematic review found that physical activity levels vary according to weather and seasonality [56]; therefore, results of this study may have differed if the accelerometer data were collected during a different season.

While accelerometers are frequently used by researchers to objectively measure physical activity levels, and are preferred over self-report measures, accelerometers are not without limitations. Accelerometers can only record movement of the body segment the device is placed on; for example, if an accelerometer is attached to a child’s wrist, it will be more likely to record non-activity movements such as twitching. This study required participants to wear the accelerometer on their hip to minimize recording of unrelated motions. Participants did not wear the accelerometer while in water (e.g., bathing or swimming). Additionally, accelerometers have difficulty recording activities performed on an incline and non-weight bearing activities (e.g., cycling); therefore, reliance on accelerometers may underestimate physical activity levels [57]. Accelerometers are also unable to provide contextual information about physical activity, such as the type of physical activity. 

## 5. Conclusions

This study is strengthened by the objective measures used for both predictor and outcome variables, thus avoiding self-report bias. Further, the present study is strengthened by its use of different sized buffers to define the neighbourhood built environment. Findings highlight the need to consider more specific neighbourhood boundaries to better characterize and understand children’s neighbourhood built environments. In particular, researchers must consider how boys and girls may have different neighbourhood ranges, and should not restrict their analyses to one size of neighbourhood for both boys and girls, if they are to better understand the relationship between environment and behaviour for children. Results also suggest that policymakers and programmers should consider developing physical activity interventions that target girls at this age to improve physical activity engagement, especially girls from low income households. Future studies should investigate the role of neighbourhood built environments on weekend MVPA to better compare and understand temporal contexts of children’s activities. Future research should also endeavor to combine GPS tracking technologies with accelerometry to investigate the different built environment contexts influencing physical activity and whether these contexts also represent opportunities for physical activity present within a child’s neighbourhood.
